# Preliminary Study of Emulsion Liquid Membrane Formulation on Acetaminophen Removal from the Aqueous Phase

**DOI:** 10.3390/membranes9100133

**Published:** 2019-10-16

**Authors:** Abdul Latif Ahmad, Zulfida Mohamad Hafis Mohd Shafie, Nur Dina Zaulkiflee, Wen Yu Pang

**Affiliations:** School of Chemical Engineering, Engineering Campus, Universiti Sains Malaysia, 14300 Nibong Tebal, Seberang Perai Selatan, Pulau Pinang, Malaysia; zulfida92@yahoo.com (Z.M.H.M.S.); nurdinazaulkiflee@gmail.com (N.D.Z.); stanley930227@hotmail.com (W.Y.P.)

**Keywords:** emulsion liquid membrane, formulation, acetaminophen

## Abstract

The aim of this study is to develop an Emulsion Liquid Membrane (ELM) system for the extraction of acetaminophen (ACTP). Firstly, ELM was formulated by the screening of liquid membrane components where the compatibility of diluent with other membrane phase components was investigated. The chosen carrier, diluent and stripping solution must comply with the reaction at the interface of the membrane to support the simultaneous processes of extraction and stripping. Therefore, parameters such as stripping agent concentration, volume ratio, initial concentration of feed phase and HCl concentration were investigated. A stable emulsion and maximum acetaminophen removal efficiency of 85% was achieved.

## 1. Introduction

The presence of contaminants in effluents and receiving waters caused by humans and animals can potentially affect the environment. These contaminants are referred to collectively as ‘contaminants of emerging concern’ (CECs) [1]. CECs can be defined using several definitions. According to U.S. Environmental Protection Agency (EPA) and U.S Department of Defence (DoD), CECs are “chemicals or materials which are characterized by a perceived, potential or real threat to human health or the environment or lack of published health standards”. They can also be perceived as “any synthetic or naturally occurring chemicals or any microorganisms that are not commonly monitored in the environment but has the potential to enter the environment and cause known or suspected adverse ecological and/or human health effects”, as defined by The United States Geological Survey (USGS). The term CECs does not necessarily correspond to newly discovered compounds in the environment due to analytical developments, but also refers to the compounds that have recently been categorised as contaminants [2]. Hence, CECs are rarely being monitored in drinking water supplies and in the environment, despite these contaminants having the potential to cause harmful ecological and human health effects, particularly on the aquatic ecosystem [3]. 

Pharmaceuticals and personal care products (PPCPs) are some of the most commonly found CECs in wastewater and in drinking water, initiated not only by humans but also through veterinary usage. The presence of pharmaceutical CECs in the environment is due to their incomplete removal during wastewater treatment or diffuse-source contamination, which are threats to both wildlife and humans. The major concern of pharmaceutical CECs is that they are usually specifically designed to target certain metabolic, enzymatic or cell-signalling mechanisms as well as maximise their biological activity at low doses. A research study by Fawell and Ong [4] stated that over 30 mg/L of pharmaceutical waste was discharged daily. Some of the most abundantly used pharmaceutical related compounds are cimetidine, diltiazem, carbamazepine, acetaminophen (ACTP), and six sulfonamide-related antibiotics. According to Al-Odaini, Zakaria [5], ACTP has the highest concentration detected among the pharmaceutical compounds tested in the Langat River in Malaysia, with a value as high as 350.3 ng/L. The same phenomenon was also noted to occur internationally such as in Spain (250 ng/L) and UK (1 µg/L). While the levels of individual pollutants are low, little is known about the long-term health implications. To make matters worse, there is some concern regarding potential ‘cocktail’ effects of different species of pharmaceutical contaminants mixed. Thus, even though these compounds existed in a trace amount and at an insignificant degree, finding an effective method to prevent further pollution of our water sources is of a major and emerging concern.

The removal of PPCPs from wastewater and drinking water is a challenging problem since there are no comprehensive methods for removing it. Removal of these pollutants in the wastewater treatment processes is generally good. Nevertheless, reports on the inability of the conventional treatment processes in wastewater treatments plants (WWTPs) to completely remove pharmaceutical contaminants in water have been well documented [6]. To some extent, the accumulated chemicals were simply discharged into the groundwater while some were not treated properly in the WWTPs [7]. This is due to the notable concentrations remaining in the final effluents owing to the relatively high influent concentrations encountered [8]. Some specific treatments have been implemented to eliminate PPCPs such as biodegradation, photocatalysis, ozonation, and the Fenton process [9]. However, some disadvantages have arisen from these methods including high investment and maintenance cost, formation of secondary pollutants, and complex operation procedures [10]. 

Therefore, integration of advanced separation technology with conventional wastewater treatments such as liquid membranes has become a great interest for many researchers. To enhance this process, liquid membrane separation was looked at in this study to be utilized in separating ACTP from contaminated water. Emulsion Liquid Membrane (ELM) which was invented by Li (1968) has shown to have promising potential for the application of ACTP extraction. Currently, ELM is introduced as an alternative technique to the separation process where it consists of three main stages, which are emulsification, extraction, and demulsification. ELM fulfils the promise of providing several attractive characteristics such as high interfacial area to volume ratio for mass transfer, economical, low energy consumption, simultaneous extraction and stripping process, high efficiency for low solute concentration, and requiring a small quantity of solvent. Additionally, it is estimated that ELM is about 40% cheaper than the conventional extraction processes [11]. With these advantages, ELM has been widely studied for industrial applications such as for the separation of various types of metal ions [12,13,14], organic compounds [15,16], and inorganic compounds [17].

Therefore, a new formulation of ELM was developed to find a suitable diluent and stripping agent for ACTP extraction. The effect of ELM formulation parameters was investigated in order to obtain its best formulation with the highest extraction efficiency. Ultrasound emulsification and Taylor-Couette Column (TCC) was used for this purpose. The influence of formulation parameters such as type of diluents, type of stripping agent, stripping agent concentration, volume ratio, and HCl concentration was investigated. The best formulation was then tested with ACTP of different initial feed concentration.

## 2. Materials and Methods

### 2.1. Materials and Chemicals

The separation process of ELM consists of three main phases (membrane, internal, and external) while the ELM itself consists of four main components (carrier, diluent, surfactant, and stripping agent). Kerosene (Sigma Aldrich, St. Louis, MO, USA), n-heptane (Merck, Darmstadt, Germany), oxylene (Merck, Hohenbrunn, Germany) [diluent] was employed to dissolve Span 80 (Merck, Hohenbrunn, Germany) [non-ionic surfactant] and trioctylamine, TOA (Merck, Darmstadt, Germany) [mobile carrier] to form the membrane phase. Span 80 was used due to its hydrophile-lipophile balance (HLB) value of 4.3 which is in the range for the application of water-in-oil (W/O) emulsifiers. Ammonia (Merck, Darmstadt, Germany), nitric acid (Merck, Darmstadt, Germany), and acetic acid (Sigma Aldrich, USA) [stripping agent] were used as the internal phase, while the external phase or model feed solution was prepared by dissolving analytical grade ACTP (Sigma Aldrich, USA) in acidic solution using HCl (Merck, Darmstadt, Germany).

### 2.2. Determination of ELM Components

The preliminary step for ELM component screening begins with a solvent extraction method. Liquid–liquid extraction was conducted to choose an appropriate and suitable diluent and stripping agent for the separation of ACTP from aqueous solution. The selection of carriers and stripping agents was considered based on the performance efficiency in ACTP extraction and was calculated using the following equation:

(1) $$\mathrm{Extraction}\;\mathrm{Efficiency},\;E\;(\%)\;=\;\frac{C_{0}-C_{f}}{C_{0}}\;\times \;100$$

where $C_{0}$ is the initial concentration of ACTP in the external phase (mg/L) while $C_{f}$ is the final concentration of ACTP in the external phase at the end of the extraction process. Extraction capacities under the ELM system of several potential diluents and stripping agents were compared. Diluent screening experiments were carried out using the three types of diluent (kerosene, n-heptane, and oxylene), while for the stripping agent screening experiments, ammonia (NH_3_), acetic acid (CH_3_COOH), and nitric acid (HNO_3_) were chosen. Complete parameters and conditions are presented in Table 1. Parameters and conditions for screening of emulsion components.

### 2.3. Diluent Screening

The diluent screening was carried out by mixing an equal volume of external phase and organic solution (a mixture of carrier and diluent) at 500 rpm for 12 h using a magnetic stirrer (IKA, Germany). The duration for the extraction process was sufficient for the phases to reach equilibrium [18]. The concentration of the diluent was fixed at 6 wt%. Then, the mixed solution was left for 15–30 min to allow phase separation of the layers by gravity settling. The final concentration of ACTP in the external phase was measured using UV-Vis (Spectroquant Pharo 300, Merck, Germany). The procedures were repeated for the different diluents.

### 2.4. Stripping Agent Screening

A mixture of external phase and organic solution was prepared according to the procedure in 2.3. An equal volume of stripping agent (0.1 M) and this mixture was mixed at 500 rpm for 12 h. The solution was then allowed 15–30 min for gravity settling to separate the phases; external phase and mix of organic solution and stripping agent. The final concentration of ACTP in the external phase was measured using UV-Vis. The procedure was repeated with the different types of stripping agent.

### 2.5. Emulsion Preparation

The ELM system was prepared via the emulsification method before being dispersed into the feed solution (external phase). In this study, the ELM was formed using the best emulsion formulation in our previous work where ultrasonicator (Telsonic Ultrasonix, Andheri, Mumbai) was used instead of mechanical agitator [19]. The diluent and stripping agent used were chosen based on the screening results in 2.3 and 2.4. The membrane phase was prepared by mixing organic membrane solution (diluent, carrier, and surfactant). The membrane phase was then mixed with the stripping agent (internal phase) at 500 rpm. These solutions were homogenized with the assistance of the ultrasonic probe (USG-150) which was equipped with a titanium horn of 3 mm diameter. The prepared emulsion was then put into a double-glass beaker where the outer glass contained cold water for cooling of the emulsification cell. The white, milky emulsion was then ready for the extraction process through ELM. The emulsion must be freshly prepared before each step of the subsequent studies. Several parameters were varied in each preparation, including stripping agent concentration (0.05, 0.1, and 0.25 M), volume ratio (2, 3, and 5 v/v%), HCl concentration (0.05, 0.1, and 0.5 M), and initial concentration of external phase (6, 8, 10, and 15 ppm).

### 2.6. Extraction Study

The external phase of ACTP feed solution was prepared by dissolving the desired amount of solute ACTP in deionized water. HCl was then added into the aqueous solution for ACTP complexes protonation [6]. The prepared W/O emulsion in 2.5 and the prepared external phase were put in the feeding container of the TCC. Both contents were mixed at a constant stirring speed for the extraction process to take place. After that, the external phase sample was taken for final ACTP concentration measurement using a UV-Vis spectrophotometer at the wavelength of 243 nm.

## 3. Results and Discussion

### 3.1. Effect of Diluent’s Type

**Figure 1:**
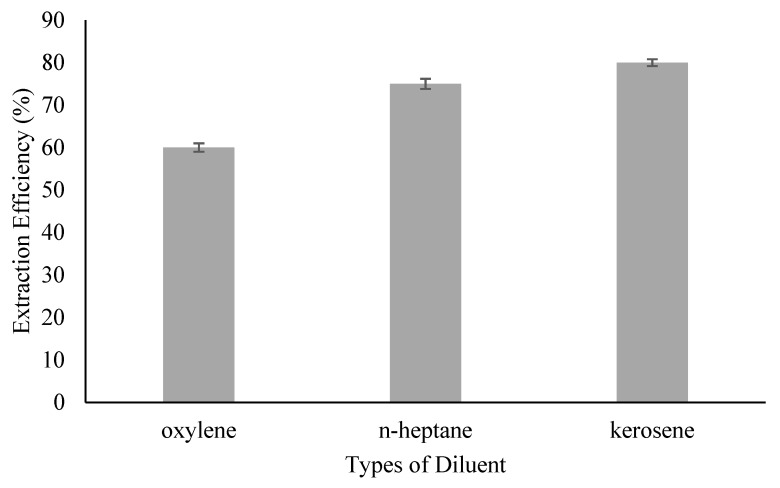
Effect of type of diluent on extraction efficiency.

During the ELM process, ACTP-TOA complex will be formed at the external-membrane interface and diffuses into the membrane-internal interphase. Hence, choice of diluent is important to ensure good formation and transport of ACTP-TOA complex across the membrane phase. Hence, the effect of the diluent’s type on the ACTP’s extraction efficiency is illustrated in Figure 1. The sequence of the extraction ability by different diluents is as follows: kerosene > n-heptane > oxylene, where the highest extraction efficiency was 82% by kerosene while the lowest was about 60% by oxylene. The extraction efficiency of the diluents is highly correlated with the polarity or dielectric constant of the diluents where lower dielectric constant increased the extraction ability: 1.8, 1.9, and 2.6 for kerosene, n-heptane, and oxylene, respectively [20]. Besides that, oxylene, an aromatic diluent, is less preferred compared to aliphatic diluents (kerosene and n-heptane) as an aromatic diluent has higher solubility in water, making it less suited for W/O emulsion formation [18,21]. 

Distribution coefficient is a ratio of concentrations of a compound in a mixture of two immiscible phases at equilibrium. The difference in solubility of the compound in these phases was calculated using the following equation:

(2) $$K_{D}\;=\;\frac{C_{i,II}}{C_{i,III}}$$

where K_D i_s the distribution coefficient, $C_{i,II}$ is the ACTP-TOA complex concentration in organic solution (mg/L) and $C_{i,III}$ is the final ACTP concentration in the external (feed) phase (mg/L). 

The distribution coefficient was tabulated as shown in Table 2. High distribution coefficient suggests that the compound is less soluble in water but more soluble in the organic solvent. The higher the distribution coefficient, the higher the permeability of the membrane to the substance. This indicates that kerosene has much better potential as a diluent than n-heptane and oxylene. In fact, the K_D_ value was almost three times more than the least feasible diluent oxylene. On the other hand, kerosene also possesses the highest boiling point and solubility with mobile carrier among the diluents tested, and relatively low toxicity and viscosity. These properties might give better stability, especially in the emulsification process of the ELM system. In general, low viscous solution results in a better emulsion stability [19,22]. Hence, kerosene will be used as the diluent for the subsequent studies.

### 3.2. Effect of Stripping Agent’s Type

**Figure 2:**
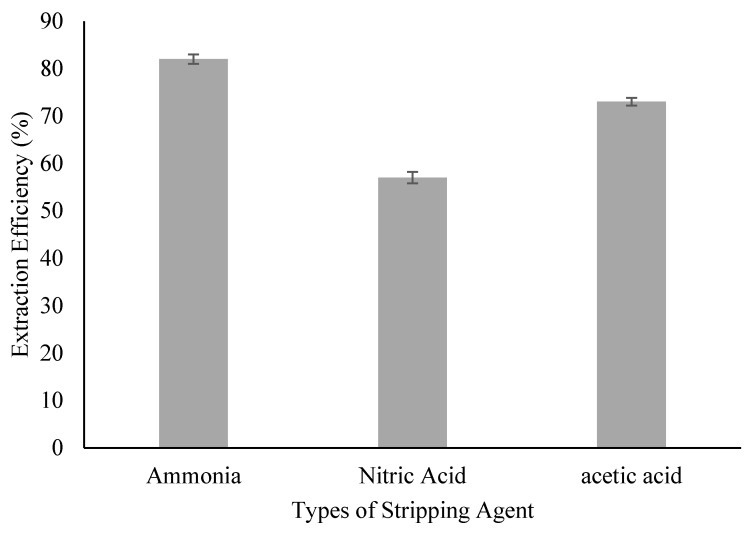
Effect of type of stripping agent on extraction efficiency.

A suitable stripping agent should be properly chosen so that it can strip out the solute (ACTP) from the solute–carrier complex effectively. The capability of NH_3_, HNO_3_, and CH_3_COOH to strip ACTP from the organic phase is presented in Figure 2. The percentage of ACTP stripped using NH_3_ was as high as 82%, compared to HNO_3_ (57%) and CH_3_COOH (73%). This shows that a basic solution is more suitable to strip ACTP from the organic phase containing TOA as mobile carrier compared to acidic solution. This condition may be possible due to the stripping reaction whereby an ACTP ion was released from the ACTP-TOA complex by the OH^−^ ion of the basic stripping solution. On the other hand, the use of an acidic stripping agent would cause incomplete ionization. The use of NH_3_ as the stripping agent was able to improve the extraction efficiency of the kerosene-diluent sample by increasing the efficiency by another 2%, although this is debatable due to the error margin. Interestingly, the use of other stripping agents reduced the extraction efficiency with a loss of up to 23% for nitric acid and 7% for acetic acid, suggesting incompatibility between these two stripping agents with the kerosene-TOA system. On the other hand, the distribution coefficient of different types of stripping solution was also evaluated as shown in Table 3. Similarly, NH_3_ possesses the highest distribution coefficient compared to others, with a K_D_ value as high as 4.55. Hence, the kerosene-ammonia system will be used for the ELM formation.

### 3.3. Effect of Stripping Agent Concentration

**Figure 3:**
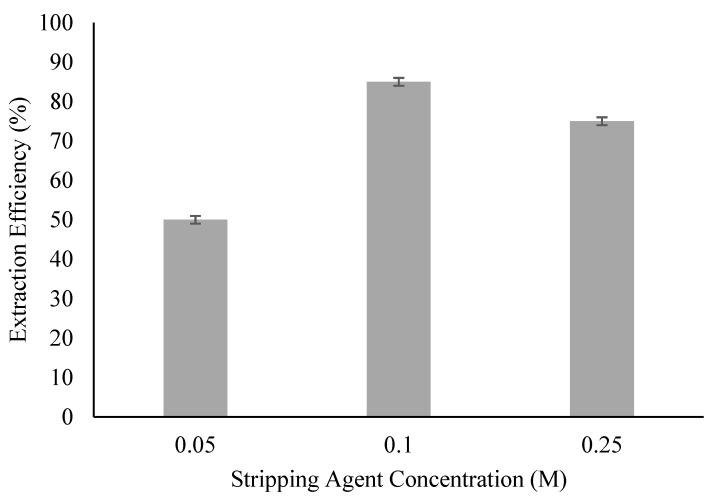
Effect of internal phase concentration on extraction efficiency. (Experimental condition: [TOA] = 6 wt%; [Span 80] = 6 wt%; Organic to Internal Ratio = 3:1; Stripping Agent = Ammonia; Diluent = Kerosene).

ELM was formed using the best diluent and stripping agent tested in the previous subsection. Nevertheless, the stripping of ACTP from the ACTP-TOA complex in ELM could still depend on the concentration of the internal phase. Figure 3 shows the effect of stripping agent concentration on the extraction efficiency of ACTP in the range of 0.05, 0.1, and 0.25 M. The results show that the increase in stripping agent concentration from 0.05 to 0.1 M resulted in an increase in the extraction performance from 50 to 85%, respectively. There was insufficient stripping agent to strip ACTP from the membrane phase at low concentration, hence the stripping was slowed down and caused saturation of the ACTP complex in the membrane phase. This is in line with the evaluation of the performance of phenol removal using ELM by [15]. According to [23], higher stripping agent concentration can strip more solute whereby more carrier molecules will be generated and freed from the complex and diffuse back to the external interface to facilitate solute extraction. The stripping solution was hence able to generate a pH gradient and thus act as a driving force for the process. This in turn will increase the extraction efficiency.

However, a further increase in concentration to 0.25 M resulted in a lower extraction efficiency than 0.1M. It is possible that reverse micelle was formed (oil-in-water, O/W emulsion) as a high concentration of the stripping agent decreases the effective number of surfactant molecules. Several studies have also reported a possible reaction between the surfactant and stripping agent which reduced extraction efficiency [24,25]. In addition, it is also possible that the high pH gradient between the internal and external phases caused a large difference in ionic strength and promoted the transportation of water into the internal phase, resulting in the emulsion swelling [26,27]. Thus, 0.1 M of NH_3_ was selected as the best stripping agent concentration in this process.

### 3.4. Effect of the Membrane to Internal Phase Volume Ratio

**Figure 4:**
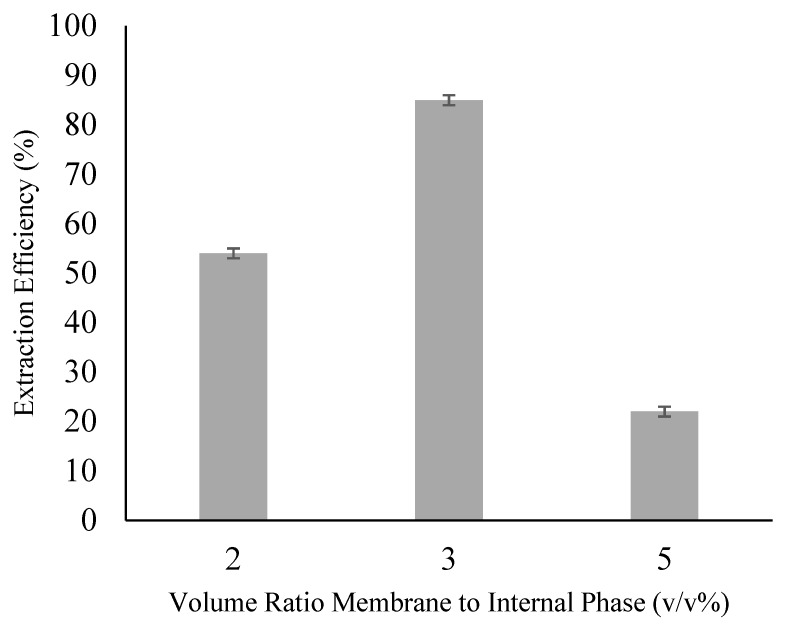
Effect of Volume Ratio Membrane to Internal Phase (Experimental condition: [Ammonia] = 0.1 M; [TOA] = 6 wt%; [Span 80] = 6 wt%; Organic to Internal Ratio = 3:1; Stripping Agent = Ammonia; Diluent = Kerosene).

The effect of the membrane to internal phase volume ratio was carried out by varying the range of volume ratio of 2:1, 3:1, and 5:1. The effect on extraction efficiency was examined as shown in Figure 4. The optimal volume ratio of membrane to internal phase was obtained at 3:1, which has the highest extraction efficiency of 85% compared to the others. A low-volume ratio of membrane to internal phase inhibits proper encapsulation of the internal phase by the organic solution thus creating emulsion with larger emulsion droplets and a thinner emulsion wall. Instability of the emulsion will be compromised and consequently, the emulsion has greater chances to break. This result is in line with [26] where high internal phase volume resulted in higher membrane breakage. The authors of [28] and [29] also observed a similar effect and better extraction at a higher treat ratio. 

While a higher volume of the membrane phase can increase the stability of the emulsion, excessive amounts of it can be counterproductive. A high-volume ratio produces a thicker emulsion wall that increases the mass transfer resistance of ACTP between the bulk external and the emulsion droplet (dispersed internal phase). High-volume usage will also affect the total operational cost of ELM. A high-volume ratio of membrane to the internal phase will also increase the surface tension of the emulsion due to the lack of surfactant molecules. Hence, the emulsion droplets are harder to be dispersed where as a consequence, larger droplets will be produced due to emulsion coalescence [30]. From this experiment, the volume ratio of 3:1 was noted to be the best volume ratio to achieve high extraction efficiency.

### 3.5. Effect of HCl Concentration in Initial Feed Solution

**Figure 5:**
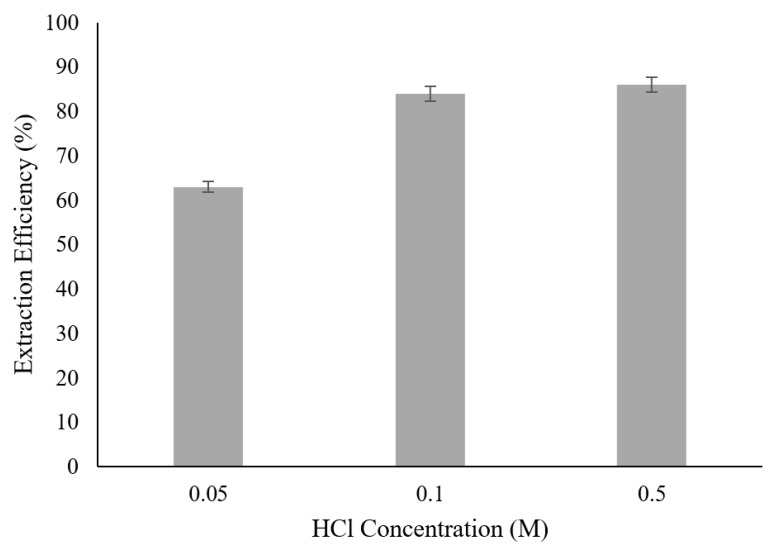
Effect of HCl concentration (Experimental condition: [Ammonia] = 0.1 M; [TOA] = 6 wt%; [Span 80] = 6 wt%; Volume Ratio = 3:1; Stripping Agent = Ammonia; Diluent = Kerosene).

External solution pH influence the complexation formation between contaminant and carrier. Strong molecule complex is needed and hence pH needs to be adjusted to a suitable value. Effect of different HCl concentration as the protonation agent for ACTP extraction was investigated and presented in Figure 5. Extraction efficiency increased as concentration of HCl increased. However, further increase in concentration beyond 0.1 M resulted in an insignificant increase of extraction efficiency. 

Lowest extraction efficiency of 63% was found at HCl concentration of 0.05 M. Lower concentration of protonation agent caused lower tendency for ACTP-TOA complex to form and hence limiting the mass transfer process. Further increase in HCl concentration of 0.5 M from 0.1 M did not significantly increase extraction efficiency; both at 86 and 84% respectively. In addition, it increases the emulsion swelling of the liquid membrane and enhanced the risk of ACTP back diffusion to external phase [31,32]. Therefore, HCl concentration of 0.1 M was chosen as the best in this study.

### 3.6. Effect of Initial Feed Concentration

**Figure 6:**
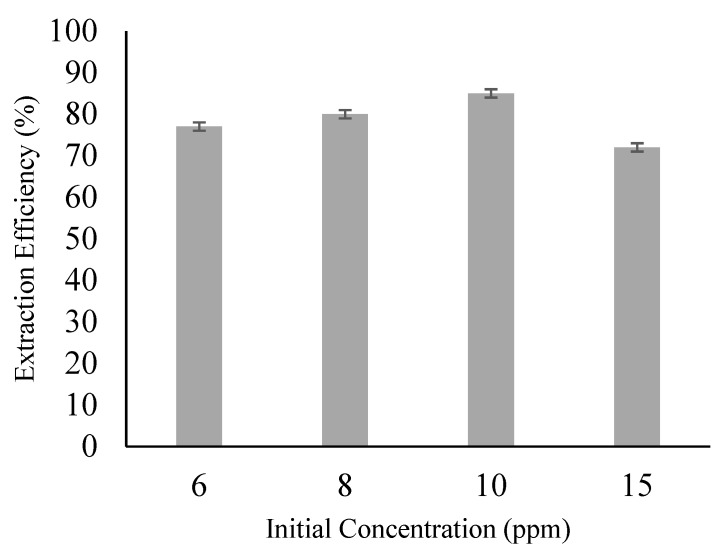
Effect of HCl concentration (Experimental condition: [Ammonia] = 0.1 M; [TOA] = 6 wt%; [Span 80] = 6 wt%; [HCl] = 0.1 M; Volume Ratio = 3:1; Stripping Agent = Ammonia; Diluent = Kerosene).

Concentration differences between the external (feed) and internal phase impose a significant driving force on the diffusion process. The extraction efficiency of ACTP was studied at initial feed concentrations of 6, 8, 10, and 15 ppm. The effect of initial feed concentration on extraction efficiency is shown in Figure 6. Extraction efficiency increased with an increase of initial feed concentration. This is due to higher chemical potential between the feed and internal phase. The highest extraction efficiency achieved was at 85% for 10 ppm ACTP. Beyond the concentration of 10 ppm, the extraction efficiency slightly decreased to 72%. When the feed ACTP concentration is low, most of the solute will diffuse into the internal phase, entering firstly through the peripheral regions of the emulsion droplets. However, at a high ppm level, since the outer layer has already been saturated, the rest of the solutes were required to permeate deeper into the emulsion droplets [33,34]. This caused stripped ACTP to pursue a longer diffusional path in the internal phase and hence decreased its extraction efficiency. This result is in line with [6] and [35].

## 4. Conclusions

ELM formulation for ACTP extraction was performed and the formulation parameters were optimised. Diluent was noted as a suitable diluent while ammonia was noted as a suitable stripping agent for this purpose. The best formulation of ELM for this study was found at 0.1 M concentration of stripping agent, volume ratio of 3:1, and 0.1 M of HCl concentration. Under the ideal conditions, around 85% of acetaminophen was successfully extracted for an ACTP initial concentration of 10 ppm.

## Figures and Tables

**Table 1:** Parameters and conditions for screening of emulsion components.

Component	Extraction	Stripping
Type of carrier	TOA	TOA
Type of diluent	Kerosene, n-heptane, oxylene	Kerosene
Type of internal phase	-	Ammonia, nitric acid, acetic acid
Initial feed solution and concentration (external phase)	10 ppm of ACTP in 0.1M HCl	ACTP-loaded organic solution
Treat ratio (v/v)	1:1	1:1

**Table 2:** Distribution coefficient of different diluents.

Diluent	K_D_
Oxylene	1.50
n-heptane	2.70
Kerosene	4.55

**Table 3:** Distribution coefficient of different stripping phase solutions.

Stripping Phase Solution	K_D_
Ammonia	4.55
Nitric Acid	1.32
Acetic Acid	2.70
